# The Predictive Value of CD3+/CD8+ Lymphocyte Infiltration and PD-L1 Expression in Colorectal Cancer

**DOI:** 10.3390/curroncol30110699

**Published:** 2023-10-31

**Authors:** Jihong Liu, Jinbang Li, Feng Luo, Shigang Wu, Bingquan Li, Kunping Liu

**Affiliations:** Department of Pathology, The Sixth Affiliated Hospital of Guangzhou Medical University, Qingyuan People’s Hospital, Qingyuan 511518, China

**Keywords:** colorectal cancer, PD-L1, microsatellite instability, CD3, CD8, prognosis

## Abstract

Aim: The immune system plays an important role in tumor development and treatment. In this study, we aimed to determine the relationships among the expressions of PD-L1, CD3, CD8, MMR proteins, clinicopathological features, and prognosis of CRC. Methods: Immunohistochemistry was used to determine the expression of PD-L1, CD3, and CD8 in 771 patients with CRC. Results: The expression of PD-L1 in TC was related to the right colon, adenocarcinoma, and dMMR, and in IC, it was related to younger CRC patients and the TNM stage. The expression of CD3 and CD8 in tumor-infiltrating lymphocytes was related to lymph node metastasis and the TNM stage. The expression of PD-L1 in TC and IC was correlated with the infiltration of CD3+ and CD8+ lymphocytes. Univariate survival analysis showed that the expression of PD-L1 in TC, IC, and dMMR was related to a better prognosis. Multivariate survival analysis showed that age, TNM stage, and dMMR were independent prognostic factors for CRC. The OS of the chemotherapy was significantly higher than that of the non-chemotherapy in III-IV TNM stage patients; CRC patients with positive PD-L1 expression in TC or IC and dMMR did not benefit from chemotherapy. Conclusions: PD-L1 expression in TC and IC was closely related to the density of CD3 and CD8 infiltration in tumor-infiltrating lymphocytes. The expression of CD3 and CD8 in tumor-infiltrating lymphocytes and the expression of PD-L1 in IC were linked to the TNM stage of CRC patients. PD-L1 expression in TC and IC and MMR status may act as an important biomarker for guiding the postoperative treatment of III-IV TNM stage CRC patients.

## 1. Introduction

Colorectal cancer (CRC) is one of the most commonly diagnosed malignancies and the leading cause of death for cancer patients in China [1]. The prognosis of CRC patients has markedly improved due to advances in surgical techniques, radiotherapy, and chemotherapy. However, the recurrence and metastasis rates in CRC patients remain high. It is necessary to further explore novel therapies for CRC treatment, and one possibility is an immunotherapeutic approach to correct abnormal immunity [2].

Immunotherapy has achieved remarkable results in the field of tumor therapy by reactivating the ability of the host immune system to kill malignant tumor cells. The most widely research are immune checkpoint inhibitors (ICIs) targeting programmed death-1 (PD-1), programmed death-ligand 1 (PD-L1), and cytotoxic T lymphocyte antigen-4 (CTLA-4), which have shown significant benefits in the treatment of advanced CRC [3]. Currently, ICIs, such as PD-1 inhibitors nivolumab and pembrolizumab, have been approved for the treatment of metastatic cancers with DNA mismatch repair defects (dMMR) and high microsatellite instability (MSI-H) [4]. The KEYNOTE-164 (NCT02460198) clinical trial confirmed the efficacy of pembrolizumab in patients with dMMR/MSI-H metastatic CRC, leading to the first entry of the era of immunotherapy for dMMR/MSI-H metastatic CRC [5]. In addition, recent studies have found that first-line use of nivolumab combined with low-dose ipilimumab has strong and long-lasting clinical benefits and good tolerance in dMMR/MSI-H metastatic CRC patients [6]. In recent years, immunotherapy has become a research hotspot in the field of tumor therapy, and various targeted immune microenvironment treatment strategies, including ICIs, have achieved certain results in CRC. However, it cannot be ignored that the efficacy of immunotherapy in CRC still needs further improvement.

The PD-1/PD-L1 axis serves as an immune checkpoint, is upregulated in many tumors and their microenvironments, and plays an important role in tumor immune escape by suppressing T helper 1 cell (Th1)-type cytotoxic immune responses [7]. The combination of PD-L1 and programmed death-ligand 2 (PD-L2) with PD-1 induces antigen-stimulated lymphocyte proliferation and the down-regulation of cytokines (such as IFN-γ and IL-2), leading to lymphocyte loss and immune tolerance [8,9,10]. Studies have reported that malignant tumors were controlled by interfering with PD-1/PD-L1 interaction to restore the ability of the innate immune system [11,12,13,14]. The detection of PD-L1 protein expression via immunohistochemistry has a certain guiding value for the treatment of ICIs [15,16,17,18]. However, the results of studies on the relationships between PD-L1 and clinical characteristics and prognosis are not fully consistent [19,20,21]. 

In recent years, the important role of the immune system in tumor development and treatment has been recognized. Cluster of differentiation 3 (CD3) and T cell receptor (TCR) form a TCR/CD3 complex that plays a key role in T cell stimulation signal transduction and T cell activation [22]. Studies have shown that CD3+ tumor-infiltrating lymphocytes have better survival rates in hepatocellular carcinoma [23]. Infiltrating cytotoxic T cells that were positive for the transmembrane glycoprotein cluster of differentiation 8 (CD8) were a prognostic factor in CRC [24]. In addition, CD8+ T cells directly attack cancer cells and play an important role in anti-tumor immunity [25,26]. However, continuous exposure to tumor antigens can lead to lymphocyte failure, lymphocyte dysfunction, and poor clinical outcomes [27,28]. Studies have shown that the expression of PD-L1 is often accompanied by the infiltration of tumor-infiltrating lymphocytes in CRC cells [16]. Other studies reported that the expression of PD-L1 on tumor cells might contribute to negative regulation against tumor-infiltrating lymphocytes in non-small cell lung cancer [29]. Deeply exploring the functional characteristics and key molecules of different types of immune cells in the immune microenvironment of colorectal cancer, discovering more therapeutic targets with clinical application value, and exploring better combination therapy strategies will help further improve the effectiveness of immunotherapy while guiding clinical physicians to provide more personalized treatment for colorectal cancer patients.

Mismatch repair (MMR) proteins play an important role in finding and repairing DNA mismatch and maintaining genome stability [30]. The abnormal function of MMR proteins is mainly caused by the deletion of MLH1, MSH2, PMS2, and MSH6. Microsatellite instability (MSI) is caused by the loss of function of DNA MMR proteins. Its molecular characteristics are considered to be important gene markers, which account for 10%~20% of all colorectal cancer cases [31]. Current research shows that the MMR protein status is related to the occurrence, prognosis, and immunotherapy of CRC [32,33]. Therefore, in the present study, we investigate the clinical relevance and prognostic significance of PD-L1, CD3, CD8, and MMR protein expression in CRC.

## 2. Material and Methods

### 2.1. Patients and Tissue Specimens

A total of 771 human primary CRC tissue specimens from 2005 to 2015 without preoperative chemotherapy, radiotherapy, and immunotherapy were collected in this study, including 734 colorectal adenocarcinomas and 37 colorectal mucinous adenocarcinomas. Of all patients, 430 were males and 341 were females, with ages ranging from 17 to 93 years and a median age of 61 years. All patients were diagnosed with CRC by two pathologists according to the World Health Organization histological tumor classification criteria. There were 79 cases with high histologic grades and 692 cases with low histologic grades. There were 363 cases with lymph node metastasis and 408 cases without lymph node metastasis. From these, 335 and 436 cases exhibited I–II TNM stages and III–IV TNM stages, respectively. Postoperative patients with III–IV TNM stage underwent 5-fluorouracil-based chemotherapy. The patients were beginning follow-up after surgery, using a combination of outpatient review, hospitalization, and telephone communication. Follow-up was performed once every six months. Complete follow-up data were obtained in 569 cases. 

### 2.2. Tissue Microarray and Immunohistochemistry

Tissue microarray (TMA) was constructed using the specimens from paraffin-embedded blocks of CRC primary tumors. Each case in the TMA was represented in 5 cores, 1 mm in size for each core. Immunoreaction for PD-L1 (SP142), CD3 (LN10), and CD8 (SP16) was purchased from Zhongshan Golden Bridge Biotechnology Co., Ltd., Beijing, China. Antigen–antibody reactions were visualized using a Ventana OptiView™ Amplification kit, followed by a Ventana OptiView™ DAB IHC Detection Kit (Ventana, AZ, USA). Counterstaining was performed using Ventana Hematoxylin II, followed by bluing reagent. Immunohistochemistry was performed using the BenchMark^®^ ULTRA (Ventana, AZ, USA). Positive and negative controls were stained concurrently and showed appropriate immunostaining.

### 2.3. Immunoreactivity Evaluation

Immunohistochemical staining of PD-L1 in tumor cells (TC) and immune cells (IC) was performed using SP142, while immunohistochemical staining of tumor-infiltrating lymphocytes was performed using CD3 and CD8. A positive (+) expression of PD-L1 was given if ≥1% of TC or IC showed convincing cell membrane staining. A negative (−) expression of PD-L1 is defined as <1% of TC or IC showing cell membrane staining. A positive (+) expression of CD3 and CD8 was given if ≥5% of tumor-infiltrating lymphocytes showed convincing cell membrane staining, while <5% of tumor-infiltrating lymphocyte stainings were negative (−). The result was judged by two pathologists who were blinded to the clinical patient data. 

### 2.4. MMR Protein Detection

MMR protein antibodies MLH1 (ES05), PMS2 (EP51), MSH2 (RED2), and MSH6 (EP49) working solutions were purchased from Zhongshan Golden Bridge Biotechnology Co., Ltd., Beijing, China. The four antibodies are all nuclear staining. Among the four antibodies, as long as one antibody staining result is negative, it is called mismatch repair deficient (dMMR), which is characterized by high-frequency microsatellite instability (MSI-H). Those with positive expression of all four antibodies were judged to be mismatch repair proficient (pMMR), which is characterized by low levels of microsatellite instability (MSI-L) or microsatellite stability (MSS).

### 2.5. Statistical Analysis

Data were analyzed using the SPSS 17.0 software package (SPSS, Inc., Chicago, IL, USA) and GraphPad Prism 5.01 (GraphPad Software, La Jolla, CA, USA). Two sets of measurement data were compared using χ^2^ test. Prognosis was estimated by the Kaplan–Meier, univariate, and Cox proportional hazard regression analysis methods. *p* values less than 0.05 were considered statistically significant. 

## 3. Result

### 3.1. Expression of PD-L1, CD3/CD8 Proteins, and MMR in 771 CRCs

**Figure 1:**
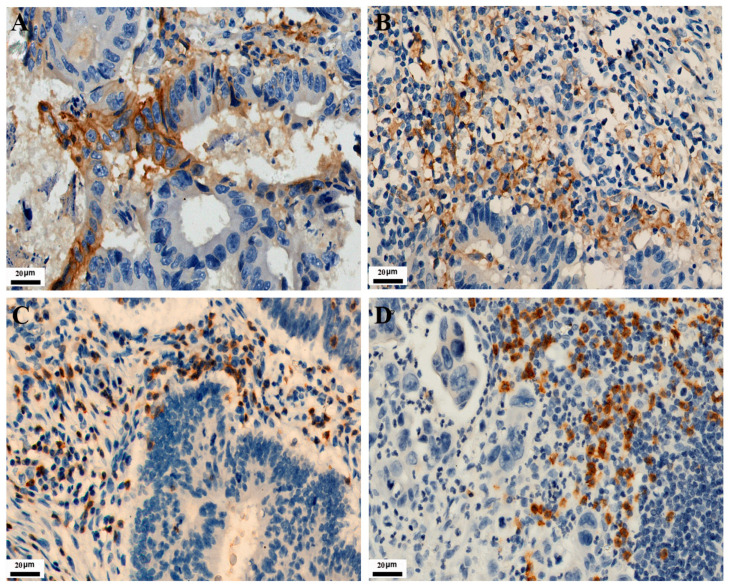
Representative images of IHC staining for PD-L1, CD3, and CD8 expression in CRC (IHC, 400×). Positive PD-L1 staining is shown in TC and IC (**A**,**B**), respectively. Positive CD3 and CD8 staining are shown in tumor-infiltrating lymphocytes (**C**,**D**), respectively.

**Figure 2:**
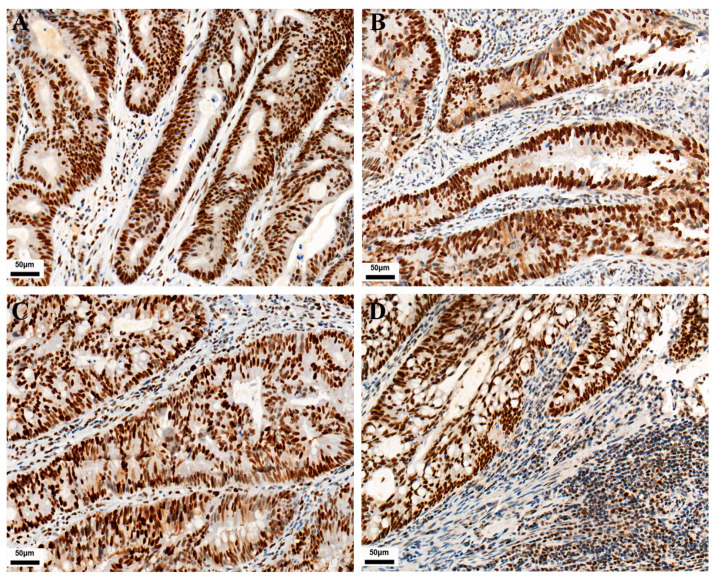
Representative images of MMR immunohistochemical staining in CRC (IHC, 200×). (**A**) MLH1 positive expression. (**B**) MSH2 positive expression. (**C**) MSH6 positive expression. (**D**) PMS2 positive expression.

As shown in Figure 1, PD-L1 expression in TC and IC is localized on the cell membrane. Among the 771 CRC cases, the positive expression number of PD-L1 in TC was 120, and the positive expression number of PD-L1 in IC was 282. Their positive expression rates were 15.6% and 36.6%, respectively. The expressions of CD3 and CD8 in tumor-infiltrating lymphocytes were localized on the cell membrane. Their positive expression number was 138 and 204, with positive expression rates of 17.9% and 26.5%, respectively. As shown in Figure 2, MMR protein expression in TC is localized on the nuclear. Immunohistochemical staining showed 93 positive cases, with an incidence of dMMR of 12.1% in the patients.

### 3.2. The Correlation of PD-L1 and CD3/CD8 Expression with MMR Proteins Expression and Clinicopathological Parameters in 771 CRCs

As shown in Table 1, PD-L1 expression in TC mostly occurred in the right colon, adenocarcinoma, and dMMR (*p* < 0.05) and had no significant correlation with patient age, gender, histological grade, lymph node metastasis, and TNM stage (*p* > 0.05). The expression of PD-L1 in IC was related to the younger CRC patients and I-II TNM stage of the tumor (*p* < 0.05) but was not significantly related to gender, tumor location, histological grade, histological type, lymph node metastasis, and MMR protein expression (*p* > 0.05). The expressions of CD3 and CD8 were significantly related to lymph node metastasis and the III-IV TNM stage (*p* < 0.05), and the expression of CD8 in the right colon is higher than that in the left colon (*p* < 0.05), while there was no significant correlation between the expression of both and age, gender, histological grade, histological type, and MMR proteins expression (*p* > 0.05). In addition, the high expression of PD-L1 in TC and IC was related to the high expression of CD3 and CD8 (*p* < 0.05), and the high expression of CD3 was also significantly related to the high expression of CD8 (*p* < 0.05).

### 3.3. The Relationship among the Expression of PD-L1, CD3, CD8, and MMR and the Prognosis of Postoperative CRC Patients

**Figure 3:**
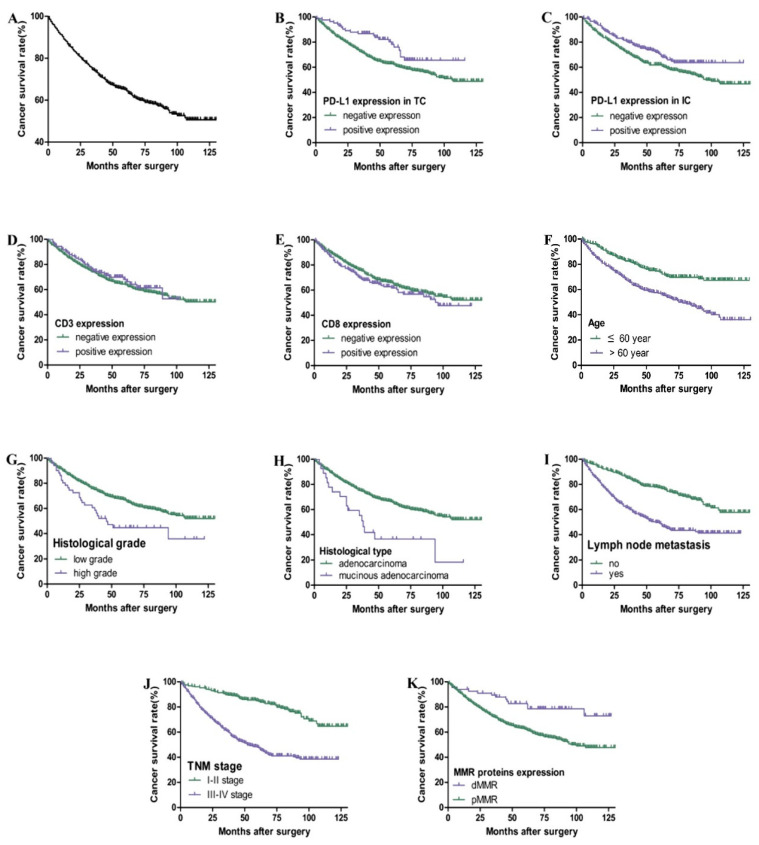
Kaplan–Meier overall survival curves of PD-L1, CD3, and CD8 expression and clinicopathological parameters in CRC patients. Overall survival rate of patients with CRC (**A**). Patients with positive expression of PD-L1 in TC (**B**) and IC (**C**) had better overall survival (*p* < 0.05). There is no significant correlation between CD3 (**D**) and CD8 (**E**) expression and prognosis (*p* > 0.05). Patients with higher age, higher histological grade, mucinous adenocarcinoma, lymph node metastasis, III–IV TNM stage, and pMMR had poor overall survival (**F**–**K**) (*p* < 0.05).

As shown in Table 2 and Figure 3, the total 3-year and 5-year survival rates for 569 cases of postoperative CRC patients are 74% and 65%, respectively. Kaplan–Meier survival curve and Cox univariate survival analysis showed that the positive expression of PD-L1 in TC (HR = 0.572), IC (HR = 0.696), and dMMR (HR = 0.412) was associated with a better prognosis. The patients with a higher age (HR = 2.091), higher histological grade (HR = 1.770), mucinous adenocarcinoma (HR = 2.247), lymph node metastasis (HR = 2.360), and III-IV TNM stage (HR = 3.503) had a worse prognosis (*p* < 0.05). However, there was no significant correlation among gender, tumor location, CD3 and CD8 expression, and prognosis (*p* > 0.05). Cox multivariate regression analysis showed that age, TNM stage, and dMMR were independent prognostic factors (HR = 2.237, 4.437, and 0.429. *p* all <0.05).

**Table 2:** Univariate and multivariate analysis of variables on overall survival in 569 CRC.

Variable	Univariate			Multivariate		
	HR	95% CI	*p* Value	HR	95% CI	*p* Value
Age	2.091	1.582–2.764	0.000 *	2.237	1.685–2.968	0.000 *
Gender	0.886	0.681–1.152	0.365	0.916	0.803–1.488	0.571
Tumor location	1.114	0.832–1.492	0.467	1.093	0.803–1.488	0.571
Histological grade	1.770	1.191–2.631	0.005 *	1.405	0.753–2.624	0.285
Histological type	2.247	1.368–3.689	0.001 *	1.321	0.609–2.864	0.481
Lymph node metastasis	2.360	1.809–3.080	0.000 *	0.774	0.531–1.128	0.182
TNM stage	3.503	2.587–4.744	0.000 *	4.437	2.898–6.792	0.000 *
CD3	0.892	0.624–1.275	0.530	0.814	0.551–1.202	0.300
CD8	1.172	0.876–1.568	0.285	1.217	0.888–1.666	0.222
PD-L1 in TC	0.572	0.365–0.897	0.015 *	0.713	0.439–1.158	0.172
PD-L1 in IC	0.696	0.520–0.932	0.015 *	0.826	0.595–1.148	0.256
MMR proteins expression	0.412	0.240–0.709	0.001 *	0.429	0.245–0.750	0.003 *

* *p* < 0.05.

**Figure 4:**
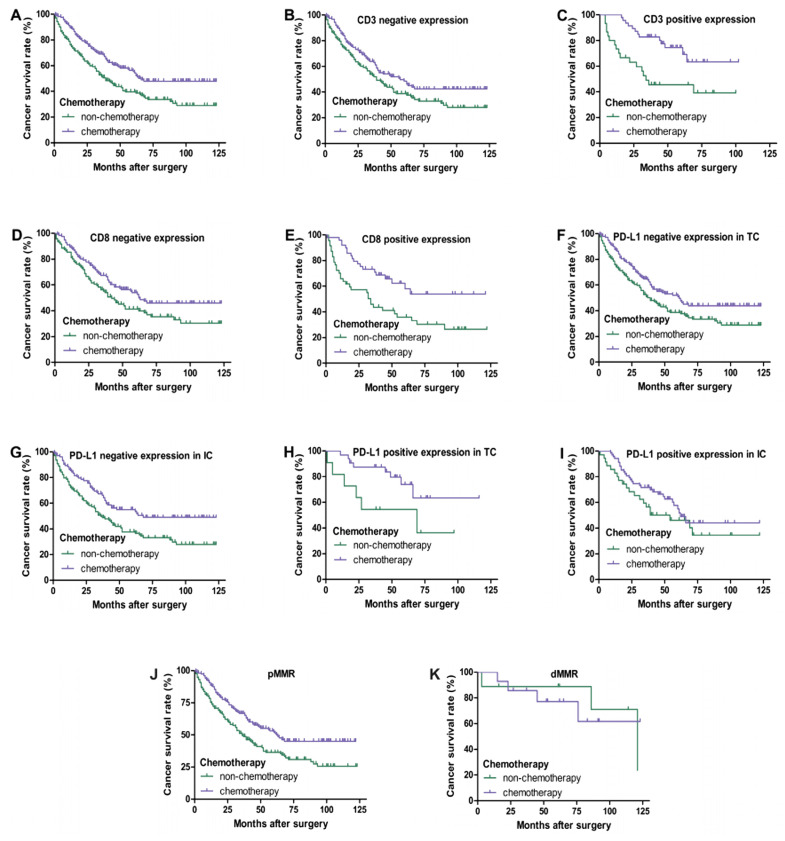
Kaplan–Meier overall survival curves of PD-L1, CD3, and CD8 expression in III–IV TNM stage CRC patients with chemotherapy. Patients with chemotherapy had a better overall survival (**A**) (*p* < 0.05). Regardless of whether CD3 and CD8 are negative (**B**,**D**) or positive (**C**,**E**) expression, patients with chemotherapy always have a better prognosis (*p* < 0.05). Patients who received chemotherapy with negative expression of PD-L1 in TC (**F**) and IC (**G**) had a better prognosis (*p* < 0.05). No significant difference in overall survival between patients with chemotherapy and non-chemotherapy of PD-L1 positive expression in TC (**H**) (*p* = 0.055) and IC (**I**) (*p* > 0.05). Patients who received chemotherapy with pMMR had a better prognosis (**J**) (*p* < 0.05). There was no significant difference in overall survival between patients with chemotherapy and non-chemotherapy of dMMR (**K**) (*p* > 0.05).

In III-IV TNM stage CRC patients, as shown in Figure 4, univariate survival analysis showed that chemotherapy improved the overall survival rate of patients (*p* < 0.05). Regardless of the positive or negative expression of CD3 and CD8 in tumor-infiltrating lymphocytes, chemotherapy significantly improved the survival rate of patients (*p* < 0.05). However, chemotherapy of patients with a positive expression of PD-L1 in TC and IC and dMMR had no significant correlation with prognosis (*p* > 0.05), while chemotherapy of patients with a negative expression of PD-L1 in TC and IC and pMMR had a better prognosis (*p* < 0.05).

## 4. Discussion

PD-L1 is expressed in a variety of tumor cells and lymphocytes of the tumor microenvironment. PD-L1 binding with PD-1 induces immune suppression and enables tumor cells to obtain immune escape; this is one of the mechanisms for tumor tolerance [34,35,36,37]. Blockade of PD1/PD-L1 signaling inhibits the immune escape of tumor cells, and the body’s own T cells play an anti-tumor role and achieve the purpose of immunotherapy [38,39]. Studies have shown that the positive expression rate of PD-L1 in TC was 12.5%, and the positive expression rate in IC was 29.8% in CRC [16]. In addition, the positive expression rates of PD-L1 in salivary duct carcinoma TC and IC were 32% and 66%, respectively [40]. In the present study, the positive expression rates of PD-L1 in TC and IC of CRC were 15.6% and 36.6%, respectively. These studies indicated that PD-L1 has different expression rates in different antibodies, and PD-L1 has a higher expression rate in tumor IC compared with TC. Our results showed that the positive expression of PD-L1 in TC was more likely to occur in right colon, adenocarcinoma, and dMMR patients. Some studies found that PD-L1 was more expressed in TC in MSI-H CRC patients than in MSS [41,42]. Inaguma et al. recently also confirmed that high PD-L1 expression in TC was significantly associated with BRAF-mutated and MMR-deficient status in CRC, typically located in the right or transverse colon [43]. Our result indicated that the expression of PD-L1 in IC was associated with young CRC patients and the I-II TNM stage. Kim et al. showed that the expression of PD-L1 in IC is related to the I-II TNM stage [16]. These findings indicated that PD-L1 expression in IC is closely linked to tumor progression in CRC.

CD3 and CD8 are important biomarkers of T lymphocytes. Many studies have reported the clinical significance and prognosis value of its expression in tumor-infiltrating immune cells of CRC. Won-Suk Lee et al. found that the positive expression rate of CD3 in tumor-infiltrating lymphocytes of CRC was 59% [44]. Huang et al. showed that the expression rate of CD8+TIL in CRC was 33% and closely related to lymph node metastasis and III-IV TNM stage [45]. In the present study, our results show that the positive expression rates of CD3 and CD8 in tumor-infiltrating lymphocytes were 17.9% and 26.5%, respectively, and both significantly correlated with lymph node metastasis and III-IV TNM stage, suggesting that CD3 and CD8 in TILs of CRC is related to tumor progression. Our results indicate that the expression of CD8 in the right colon is higher than that in the left colon. Zhang et al. observed [46] that compared with left-side CRC, the infiltration of CD8+T cells in right-side CRC was enhanced, which is consistent with our research results. It has been reported that in some cases, right CRC and left CRC exhibit various biological and clinical differences, including embryonic origin, microbiota load, vascular supply, and main physiological functions [47,48], which may affect somatic mutations and immunophenotypes between two different disease locations, thereby affecting the selection of molecular targeted drugs or immunotherapy methods.

Our study showed that the expressions of PD-L1 in TC and IC are both correlated with the expression of CD3 and CD8 in tumor-infiltrating lymphocytes. Tomohiro Kikuchi et al. identified a subpopulation of patients with pMMR-CRC who were positive for PD-L1in TC and who had levels of elevated CD8+ and CD4+ TILs infiltration similar to those dMMR-CRC patients [49]. Kim et al. found that PD-L1 expression in TC and IC of CRC were accompanied by increased infiltration of CD3+ TILs [16], and Wang et al. showed that the expression of PD-L1 in TC of CRC was positively associated with CD8 + TILs density [50]. In summary, the high expression of PD-L1 in TC and IC is accompanied by increased expression of CD3 and CD8 in tumor-infiltrating lymphocytes. This may partly be due to the fact that activated T cells can secrete cytokines such as interferon-γ, thereby inducing the expression of PD-L1 in tumor cells and TILs in the immune microenvironment [51].

Previous studies found that the relationship between PD-L1 expression and prognosis in CRC is controversial and not fully consistent. Zhu et al. found that the low expression of PD-L1 in TC has better overall survival in CRC [21]. Conversely, Li et al. [19] found that higher expressions of PD-L1 in TC correlate with better prognosis of CRC patients. Also, the results of Soo Jung Lee et al. showed that patients with high expression of PD-L1 in IC have better prognosis for colon cancer [52]. In the present study, we found that the positive expressions of PD-L1 in TC and IC were associated with better prognosis, where PD-L1 expression in IC was found to play an important role in tumor immune escape and influence tumor progression and was thereby correlated with a favorable prognosis [52]. In our study, no significant correlation was found between the positive expression of CD3 and CD8 in CRC patients and prognosis. Nosho et al. found that in the multivariate model, CD3+ and CD8+ cell density was not associated with survival in CRC [53]. In addition, our findings showed that dMMR patients had a better prognosis than pMMR patients, which was consistent with the result reported by Qin et al., who reported that colon cancer displaying dMMR had a better 5-year overall survival (OS) rate and disease-free survival (DFS) rate than the patients with pMMR [54]. Our results suggest that the prognosis of CRC patients with a positive expression of PD-L1 in TC is better than the negative, which may partly be attributed to the positive expression of PD-L1 in TC being more likely to occur in dMMR as well as the positive expression of PD-L1 in IC significantly correlating to the early TNM stage. 

5-Fu and its derivatives are commonly used in the chemotherapy of CRC. In the present study, we evaluated the predictive value of CD3/CD8/PD-L1 protein expressions of CRC in adjuvant chemotherapy. Being consistent with Li et al.’s results [55], our study also found that chemotherapy significantly improved the survival time of III-IV TNM stage CRC patients. Patients with negative expression of PD-L1 in TC or IC and pMMR benefit from the chemotherapy. Our results show whether the positive expression of CD3 and CD8 in tumor-infiltrating lymphocytes does or not does not affect the chemotherapy effect of CRC patients. However, CRC patients with positive PD-L1 expression in TC or IC and dMMR did not benefit from chemotherapy. For CRC patients with positive PD-L1 expression and dMMR status, immune checkpoint inhibition may be a potentially more effective treatment strategy [33,42,56]. Therefore, our results suggest that the expression of PD-L1 and MMR status may act as an important biomarker for guidance in the postoperative treatment of CRC patients with the III-IV TNM stage.

In conclusion, PD-L1 expression in TC and IC was closely related to the density of CD3 and CD8 infiltration in tumor-infiltrating lymphocytes. The expression of CD3 and CD8 in tumor-infiltrating lymphocytes and the expression of PD-L1 in IC were linked to the TNM stage of CRC patients. PD-L1 expression in TC and IC and MMR status may act as an important biomarker for guidance postoperative treatment of III-IV TNM stage CRC patients.

## Figures and Tables

**Table 1:** Expression of PD-L1, CD3, and CD8 and their correlation with clinicopathological parameters and MMR status in 771 CRC.

Variable	Cases	PD-L1+ in TC	*p*	PD-L1+ in IC	*p*	CD3+	*p*	CD8+	*p*
Age									
≤60	351	62 (17.7%)	0.142	142 (40.5%)	0.041	71 (20.2%)	0.123	103 (29.3%)	0.097
>60	420	58 (13.8%)		140 (33.3%)		67 (16.0%)		101 (24.0%)	
Gender									
Male	430	65 (15.1%)	0.700	156 (36.3%)	0.848	83 (19.3%)	0.254	116 (27.0%)	0.714
Female	341	55 (16.1%)		126 (37.0%)		55 (16.1%)		88 (25.8%)	
Tumor location									
Left colon	574	78 (13.6%)	0.010 *	210 (36.6%)	0.993	95 (16.6%)	0.096	132 (23.0%)	0.000 *
Right colon	197	42 (21.3%)		72 (36.5%)		43 (21.8%)		72 (36.5%)	
Histological grade									
Low grade	692	104 (15%)	0.225	255 (36.8%)	0.640	118 (17.1%)	0.069	179 (25.9%)	0.270
High grade	79	16 (20.3%)		27 (34.2%)		20 (25.3%)		25 (31.6%)	
Histological type									
Adenocarcinoma	734	119 (16.2%)	0.020 *	274 (37.3%)	0.053	131 (17.8%)	0.868	194 (26.4%)	0.936
Mucinous adenocarcinoma	37	1 (2.7%)		8 (21.6%)		7 (18.9%)		10 (27.0%)	
Lymph node metastasis									
No	408	65 (15.9%)	0.766	160 (39.2%)	0.107	43 (10.5%)	0.000 *	91 (22.3%)	0.006 *
Yes	363	55 (15.2%)		122 (33.6%)		95 (26.2%)		113 (31.1%)	
TNM stage									
I-II	335	56 (16.7%)	0.439	137 (40.9%)	0.029 *	34 (10.1%)	0.000 *	76 (22.7%)	0.037 *
III-IV	436	64 (14.7%)		145 (33.3%)		104 (23.9%)		128 (29.4%)	
CD3 expression									
Low	633	77 (12.2%)	0.000 *	188 (29.7%)	0.000 *	\		135 (21.3%)	0.000 *
High	138	43 (31.2%)		94 (68.1%)				69 (50.0%)	
CD8 expression									
Low	567	71 (12.5%)	0.000 *	166 (29.3%)	0.000 *	\		\	
High	204	49 (24.0%)		116 (56.9%)					
MMR proteins expression									
*pMMR*	678	98 (14.5%)	0.022 *	246 (36.3%)	0.649	124 (18.3%)	0.445	184 (27.1%)	0.248
*dMMR*	93	22 (23.7%)		36 (38.7%)		14 (15.1%)		20 (21.5%)	

* *p* < 0.05.

## Data Availability

Data generated for the current study are available from the corresponding author upon reasonable request.

## Abbreviations

CD3: cluster of differentiation 3; CD8: cluster of differentiation 8; CRC: colorectal cancer; IHC: immunohistochemistry; TC: tumor cells; IC: immune cells; PD-L1: programmed death-ligand 1; PD-1: programmed death-1; Th1: T helper 1; ICIs: immune checkpoint inhibitors; MMR: mismatch repair; MSI: microsatellite instability; TMA: tissue microarray; dMMR: mismatch repair deficient; pMMR: mismatch repair proficient; MSI-H: high-frequency microsatellite instability; MSI-L: low levels of microsatellite instability; MSS: microsatellite stable; OS: overall survival.
